# Practical Notes on Diagnosis and Treatment

**Published:** 1907-04-13

**Authors:** 


### Practical Notes on Diagnosis and Treatment.

**Erysipelas.**

The chief characteristic of erysipelas is to invade fresh tissue by rapidly spreading at the margin; this feature is not found in any other similar affection, and it is of great value in diagnosis.

**Syphilis of the Lung.**

The physical signs in this condition closely resemble those of chronic tubercle. The diagnosis depends on (*a*) absence of tubercle bacilli from the sputum; (*b*) history of infection; (*c*) other evidences of syphilis; (*d*) the result of anti-syphilitic treatment.

**Pleural Effusion.**

When the fluid in a case of serous effusion has been drawn off the application of an ice-bag over the affected side, and continued for some days, always tends to hinder the fresh secretion of fluid. It is also an advantage to encourage the patient to take deep draughts of air into the lungs, as this promotes the expansion of the lung which had been compressed by the fluid effusion.—*Dr. Lees.*

**Site of Spinal Lesion in Paraplegia.**

Every compression or disease affecting a transverse section of the spinal cord above the lumbar enlargement has a tendency to increase the activity of the reflexes in the lower limbs. When the lesion is at the level of the lumbar enlargement the reflexes are lost, as the centres commanding the reflex arcs upon which the tendon jerks depend are then destroyed.

**Concussion of the Brain.**

The symptoms generally attributed to concussion are due, not to the concussion itself, but to contusion of the brain or to extravasation of blood. Should symptoms of intracranial irritation or inflammation show themselves they should be dealt with actively. In the early stage the application of cold to the head by means of Leiter's tube is the most efficient local, and free purgation the most effective general, means, with a very low diet. If the inflammatory action is great, a free bleeding from the jugular vein or from the arm is to be advocated, and this operation may in many cases be repeated with much advantage. In chronic cases the value of mercury, taken internally, cannot be doubted.—*Mr. Bryant.*

**Acute Inflammation of Middle Ear.**

The first step in treatment is to abstract blood from the ear. This is best effected by the application of one, two, or three leeches, according to the age of the child. A piece of cotton wool should be placed in the external meatus to prevent the entrance of blood. The leeches should be applied, if possible, inside the concha. If this is impracticable they should be placed in front of the ear or over the mastoid. After the bleeding has ceased the cotton wool should be removed, and water, as hot as can be borne, should be gently injected into the external meatus at frequent intervals. In the way of medicines, bromide of potassium and tincture of aconite are of great service.—*Mr. Woakes.*

**Pruritus Ani.**

The following has been recommended as almost a specific in cases of pruritus ani:—Take of sulphate of zinc and alum equal parts; heat the two until all the water of crystallisation has been driven off, and dissolve the residue in water (ʒj. to ℥j.). The solution must be applied freely to the site of irritation.

**Vague Pains.**

Never hastily pronounce a case of vague pains to be "neuralgia," "rheumatism," or the like. Care, and especially prolonged observation, will usually guide you to a correct conclusion, and will save you from the grave discredit of attributing to a trivial cause such serious maladies as bone disease, tumours, or internal aneurism.—*Mr. Marmaduke Shield.*

**Asthma.**

The inhalation of pyridin, 10 to 20 drops placed on a pocket-handkerchief, will often give prompt relief. One of the most certain remedies is morphine by hypodermic injection; but in long-standing cases, with a dilated and feeble heart, this treatment is by no means free from risk.

**Pain in Hæmorrhoids.**

Uncomplicated piles are never the source of acute pain. This is a useful clinical fact, as it will compel you to search for some special complication in every case in which a patient, the subject of piles, complains of acute suffering. The common causes of such pain are thrombosis, prolapse, strangulation, and fissure.—*Mr. Pearce Gould.*

**Skene's Glands.**

Catarrhal inflammation of Skene's glands is one of the causes of "painful sitting." It also causes pain during coition, and may be associated with painful micturition. Hot sitz baths may give relief, but sometimes it is necessary to apply iodine or carbolic acid in glycerine to the interior of the glands, the orifices of which are close to the urethral opening.

**Leucoplakia and Cancer.**

A case of leucoplakia is more liable than any other tongue to have cancer developed in it, although such a result is not by any means a certainty. I would not remove a tongue simply because it had leucoplakia, but I should warn the patient that he must be very careful, and the moment any growth showed itself he ought to have his tongue removed.—*Mr. Christopher Heath.*

**Opium in Cardiac Disease.**

In mitral and other forms of heart disease which are constantly accompanied in their later stages by distress and cough, restlessness and sleeplessness, opium is of great service. Many deprecate its use in these conditions on account of its action on the respiratory centre; but an extensive experience has taught me that it may be administered without fear if given in moderate doses by the mouth, as by this method the shock and depressant action on the heart, which render the use of the hypodermic method dangerous in such cases, are avoided.—*Dr. Cheadle.*

